# Challenges in the humanized mouse model for cancer: A commentary

**DOI:** 10.46439/cancerbiology.2.022

**Published:** 2021

**Authors:** Eric Ramirez-Salazar, Meenhard Herlyn, Rajasekharan Somasundaram

**Affiliations:** The Wistar Institute, 3601 Spruce Street, Philadelphia, PA, USA

**Keywords:** Humanized mouse model, Tumor microenvironment, Mast cells, Therapy resistance

## Abstract

The complexity of the tumor microenvironment has been a challenge for understanding the mechanisms of therapy resistance. The development of improved animal models that closely mimic human disease is key for understanding and treating diseases. Recently, a new humanized mouse model has been developed that enables the study of human immune cells in tumor host-cell interactions. In this commentary we highlight the critical aspects of mast cells in immune therapy resistance. These mast cells release cytokines that downmodulate HLA class I on the malignant cells making them inaccessible the cytotoxic activity of T cells.

## Introduction

A tumor mass consists not only of cancer cells but also a variety of resident and infiltrating host cells, extracellular matrix proteins, and secreted factors, that collectively are known as the tumor microenvironment (TME). Progression from non-aggressive to aggressive tumors is influenced by interactions of cancer cells with TME that largely determine whether the tumor is eradicated, metastasizes, or organizes as dormant micro-metastasis [1]. The TME is also involved in the response and resistance to treatment. This makes it important to investigate in-depth the functional roles of components of the TME. Therapy resistance to checkpoint inhibitors is closely related to the infiltration of T cells into the TME, and this is mediated by several mechanisms. However, the mechanism of resistance to immune-based therapies are often unclear due to the lack of models of study that replicate the human TME. Therefore, the development of animal models is needed that allow mimicking the human environment as closely as technically feasible.

## Discussion

In a recent publication, Somasundaram et al. [2], characterized a humanized mouse model for the study of the melanoma TME. Highly immune-deficient NSG mice were grafted with human fetal thymus and injected with autologous liver-derived CD34+ cells. Due to the endogenous expression of six human cytokines, the hematopoietic stem cells differentiated to a variety of innate and specific immune cells, including CD4^+^ and CD8^+^ T cells, B cells, monocytes, and mast cells. The human immune cells emerged after approximately 8 to 10 weeks when ~20% of the white blood cells were of human origin with the percentage increasing over time to >80%. There was little or no indication of graft-versus-host-disease symptoms. By 12–14 weeks after CD34^+^ cells administration, when mice reached physiological levels of t human B- and T-cells, the mice were challenged with HLA-A-allele matched human melanoma cells. Due to the emerging immune response, growth of the human tumors was slowed by 50% at most but in none of the >15 experiments did the spontaneous immune response eradicate the tumors. When the animals were treated with anti-PD1 checkpoint inhibitor antibodies, growth was further reduced by 40%−60% suggesting that the anti-tumor response was significantly stimulated but again, there were no complete responses.

The authors then searched for mechanisms of the incomplete eradication of tumors using RNAseq and multiplex immune histochemistry. The data suggest a key role for mast cells in therapy resistance in melanoma. The presence of tumor-infiltrating mast cells and the co-localization of FOXP3-positive cells are a likely mechanism of localized downregulation of HLA class I molecules by the tumor cells. The downmodulation of HLA class I was associated with poor infiltration of Granzyme-producing CD8^+^ cells, and this may be inducing a mechanism of tumor cell escape and evolving resistance to continued therapy. This conclusion was supported by the depletion of mast cells, which are strongly expressing the receptor tyrosine kinase c-Kit and are thus susceptible the kinase inhibitors sunitinib or imatinib. The combination of anti-PD1 antibodies and kinase inhibitors sunitinib or imatinib regressed the tumors, which remained undetectable throughout the duration of the experiments. These experiments suggest that mast cells, through secretion of cytokines such as interferon gamma-induced protein 10 (IP-10), also known as CXCL10, induce local downmodulation of HLA class I expression, thus rendering the tumor cells undetectably by cytotoxic T cells.

It is important to highlight that despite the considerable progress demonstrated by this mouse model, there is no perfect model. One limitation is due to the cross-reactivity, or lack thereof, of cytokines and growth factors between species, resulting in inadequate development and differentiation of cellular subsets [3–5]. Another weakness of the model, besides the use of human fetal tissues, is the incompatibility in the minor HLA B-D alleles. Fully matching those would be very difficult even impossible despite the availability of several hundred of cell lines and patient-derived xenografts. Still, we did not graft-versus-graft immune responses.

The animal model presented by Somasundaram et al. [2] differs from those using transgenic mice by the concentration levels reached of the cytokines and growth factors in circulation. In this model, cytokines and grow factors are delivered by viral and plasmid vectors. The concentrations of the cytokines IL-3, IL-7, and the grow factor GM-CSF stimulated the production of CD45^+^ cells in circulation and expression of SCF, FLT3, and THPO by plasmid vectors improved T-cell and myeloid differentiation. In this way, the mouse model is avoiding side effects due to the high levels of transgenes.

Other humanized mice models generated without transplantation of human thymic tissue are deficient in the human major histocompatibility complex-derived T-cell development. To solve this, Somasundaram et al. [2], transplanted human thymus onto the mice by sub-renal capsule engrafting. This strategy of cografting stem cells and thymus tissues accelerated the differentiation of stem cells to T cells.

## Conclusion

The development of immunotherapy in patients with most malignancies has increased the need for mouse models with immune competent systems. The model presented here provides one step closer to the ultimate goal of completely mimicking the conditions in humans.

In Memoriam of Rajasekharan Somasundaram, Ph.D., 1956–2021.
